# Identification of Drug Combinations Containing Imatinib for Treatment of BCR-ABL+ Leukemias

**DOI:** 10.1371/journal.pone.0102221

**Published:** 2014-07-16

**Authors:** Yunyi Kang, Andrew Hodges, Edison Ong, William Roberts, Carlo Piermarocchi, Giovanni Paternostro

**Affiliations:** 1 Sanford-Burnham Medical Research Institute, La Jolla, California, United States of America; 2 Salgomed Inc., Del Mar, California, United States of America; 3 Rady Children's Hospital, Department of Pediatrics, University of California San Diego, San Diego, California, United States of America; 4 Department of Physics and Astronomy, Michigan State University, East Lansing, Michigan, United States of America; Emory University, United States of America

## Abstract

The BCR-ABL translocation is found in chronic myeloid leukemia (CML) and in Ph+ acute lymphoblastic leukemia (ALL) patients. Although imatinib and its analogues have been used as front-line therapy to target this mutation and control the disease for over a decade, resistance to the therapy is still observed and most patients are not cured but need to continue the therapy indefinitely. It is therefore of great importance to find new therapies, possibly as drug combinations, which can overcome drug resistance. In this study, we identified eleven candidate anti-leukemic drugs that might be combined with imatinib, using three approaches: a kinase inhibitor library screen, a gene expression correlation analysis, and literature analysis. We then used an experimental search algorithm to efficiently explore the large space of possible drug and dose combinations and identified drug combinations that selectively kill a BCR-ABL+ leukemic cell line (K562) over a normal fibroblast cell line (IMR-90). Only six iterations of the algorithm were needed to identify very selective drug combinations. The efficacy of the top forty-nine combinations was further confirmed using Ph+ and Ph- ALL patient cells, including imatinib-resistant cells. Collectively, the drug combinations and methods we describe might be a first step towards more effective interventions for leukemia patients, especially those with the BCR-ABL translocation.

## Introduction

The BCR-ABL fusion protein results in the Philadelphia chromosome and is present in 95% of chronic myeloid leukemia (CML) patients and 20–40% of adult acute lymphoblastic leukemia (ALL) patients. [1]–[3] The BCR-ABL translocation is characterized by a constitutively active fusion tyrosine kinase and plays a causal role in CML. It is therefore an attractive target for drug therapy. [4], [5] Imatinib mesylate (Gleevec/Glivec), a selective Abl kinase inhibitor, has been shown to have marked efficacy as a single agent and is used as a first-line therapy for the treatment of CML and Ph+ ALL patients. [6]

Despite the remarkable hematologic and cytogenetic responses to imatinib, a substantial number of patients develop resistance, especially in advanced cases, leading to disease relapse. [7] Resistance to imatinib has been attributed to secondary genetic mutations in the target and to downstream mechanisms. [8] In addition, imatinib is unable to eradicate the leukemic blast cells, thus only inducing remission. [7] To circumvent these problems, many research groups have investigated combinatorial drug regimens with drugs that have different modes of actions. [9]–[11] Most of the combinatorial studies, however, considered combinations of two or three drugs, which may not suffice to achieve complete inhibitory action on multiple oncogenic pathways. [12]

## Results

### Search flow for optimization of combinatorial drugs

**Figure 1:**
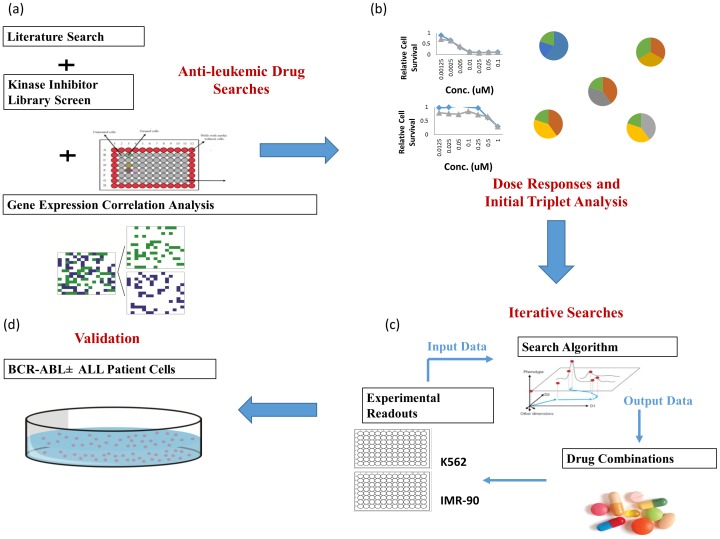
Scheme of our search strategy (a) anti-leukemic drugs were selected using three approaches: kinase inhibitor library screens, correlation analysis, and literature survey. (b) Dose responses of single agent and pair-wise analysis with a fixed dose of imatinib (pairs and triplets) were performed. (c) The drug combinations were optimized using iterative search algorithm (d) The optimal drug combinations were validated with primary patient cells.

A scheme of our search strategy to identify optimal drug combinations is illustrated in Figure 1. We first selected 11 drugs to be combined with imatinib and then used the BCR-ABL+ K562 cell line as a model system. The drug combinations were then optimized using iterative loops that process input data from experimental readouts. We applied an algorithm suggesting combinations to be tested at each iteration.

Dose responses of single agents were performed to determine the starting doses to be used. We then performed a pair analysis, in which all the possible pair combinations of agents were studied; indicated in the Figure 1 as “triplets” because imatinib was also added, at fixed dose. The data from the pair+imatinib analysis (triplets) were fed into a model that estimates the effectiveness of large drug combinations, assuming only pairwise interactions among compounds. The top combinations predicted by this model were then used in the iterative loop as initial conditions for the experimental search. At each iteration new combinations were generated and experimentally tested, and this was repeated until effective drug combinations were identified. The best combinations identified using the K562 cell line were validated using primary cells from BCR-ABL+ ALL patient xenografts and ALL patients.

### Selection of anti-leukemic drugs

**Figure 2:**
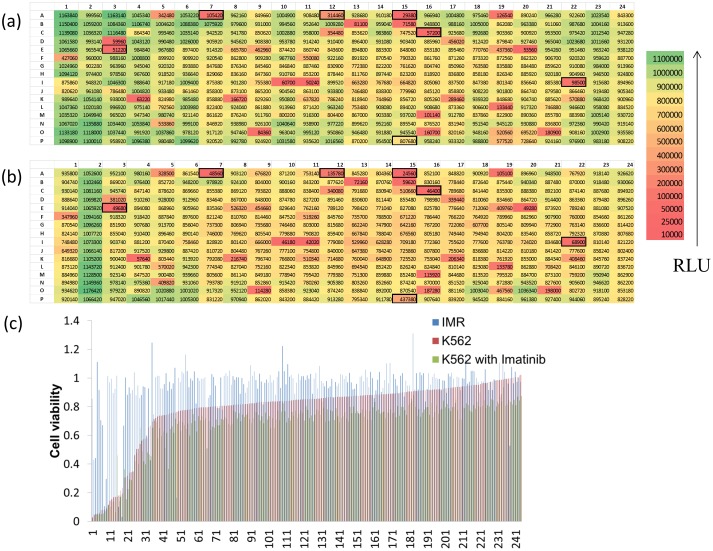
Selection of anti-leukemic drugs from kinase inhibitor library screens (a) A representative screening plate of a kinase inhibitor library consisting of 244 inhibitors. The ATP content of K562 cells (a measure of cell viability) was measured and the luminescence intensity of each well was color-coded according to the scale presented (RLU, relative luminescence unit). The corresponding compound ID in the plate map is shown in Table S1. (b) A representative screening plate of a kinase inhibitor library treated with 0.125 µM imatinib. The kinase inhibitors selected from the screens are indicated with black borders. (c) The kinase inhibitors are rank-ordered from the lowest to the highest cell viability in K562 cells without imatinib (red bars). The corresponding viability of IMR and K562 cells with imatinib are indicated using blue and green bars. Several drugs are selectively killing K562 cells.

We employed three different methods to select the set of 11 anti-leukemic drugs that are studied with imatinib in the combinatorial drug searches. We first screened an EMD kinase inhibitor library that consists of 244 kinase inhibitors with/without imatinib (Figure 2). The cell viabilities were measured by ATPlite assays in the leukemic cell line (K562) and in a control fibroblast cell line (IMR-90) [13], [14] 72 hours after the treatment with the kinase inhibitors. We have rank-ordered the kinase inhibitors according to the selectivity index, which is defined as the ratio v_n_/v_c_, where v_n_ and v_c_ indicate the viability of IMR-90 and K562. Four highly ranked kinase inhibitors were selected based on the analysis; A15, C16, E3, I22 (Table S1). We also selected three kinase inhibitors that showed synergistic or additive effects when tested with 0.125 µM imatinib based on the imatinib combination index (v_c_/v_imatinib_, where v_c_ and v_imatinib_ indicate the viability of K562 without/with imatinib); A07, A12, P15 (Table S1). This fixed dose of imatinib was chosen so that it gives only 10–20% cell killing according to the dose response curve of imatinib.

In addition, we chose ABT263, Axitinib, and 17AAG based on a correlation analysis between drugs and imatinib responses. The correlation signs were derived from the Pearson correlation between mRNA expression and drug IC_50_ values on cancer cells data from Garnett *et al*. [15] The imatinib-17AAG pair was among the top scoring negative-positive and positive-negative pair set, whereas ABT263 and Axitinib were among the top of the positive-positive and negative-negative pair sets (Table S2). The rationale for this analysis was to identify drugs that might either share gene expression correlations (the positive-positive and negative-negative group) with imatinib or that might have opposite correlations (the negative-positive and positive-negative set). We hypothesized that the first type of compounds might act on similar pathways, via different targets, and therefore directly reinforce the effects of imatinib, while the second type might ideally complement it. These strategies should counteract resistance mechanisms of different types.

We also added the mitogen-activated protein kinase (MEK) inhibitor PD0325901 to the drug list based on literature data. [16], [17] The list of the small molecules used for our combinatorial study and their targets are shown in Table 1.

**Table 1:** The list and targets of the drugs used in the combinatorial studies.

Drug name	Well ID	Pubchem ID	Drug Targets	Target family	Effector pathway/biological process
Akt Inhibitor IV	A07	5719375	AKT	AGC	Apoptosis, Metabolism, PI3K/MTOR
Alsterpaullone, 2-Cyanoethyl	A12	16760286	CDK1, GSK3B, CDK5	CMGC	Mitosis
PDK1/Akt/Flt Dual Pathway Inhibitor	A15	5113385	PDK1/AKT/FLT	AGC, ATYPICAL, TK	PDK1/AKT/FLT
Cdk1/2 Inhibitor III	C16	5330812	CDK1/2	CMGC	Cell Cycle
EGFR Inhibitor	E03	9549299	EGFR	TK	ERK Signalling, PI3K/MTOR
JNK Inhibitor IX	I22	16760525	JNK	CMGC	Stress Pathways
WHI-P180, Hydrochloride	P15	5687	CDK2	CMGC	Cell Cycle
ABT263	N/A	24978538	BCL2, BCL-XL, BCL-W	Other	Apoptosis
Axitinib	N/A	6450551	PDGFR, KIT, VEGFR	RTK	Angiogenesis, ERK Signalling, PI3K/MTOR
17AAG	N/A	6505803	HSP90	Other	Other
PD0325901	N/A	9826528	MEK	STE	ERK Signalling
Imatinib	N/A	5291	ABL, KIT, PDGFR	CTK, RTK	Cytoskeleton, ERK Signalling, PI3K/MTOR

### Dose responses and pair analysis

Dose responses for the selected small molecules were measured over a 100–fold concentration range (seven-points, as shown in Figure S1). The IC_50_ values for most of the drugs ranged between 0.1 and 1 µM although I22 and 17AAG showed 10-fold lower values of IC_50_ (Table S3). Additional dose responses in combination with imatinib confirmed the additive effects of the three drugs we chose from the initial screening (A7, A12, P15). We also detected an additive effect of A15 at low doses. ABT263, selected from the correlation analysis, also exhibited additive effects. For the subsequent pair analysis, the doses that cause a K562 cell killing between 20–30% in the presence of imatinib were chosen as high doses whereas those with killing between 10–20% were defined as low doses. These doses were selected in order to avoid that the cytotoxicity of single agents would dominate the response to the combinations. The full triplet analysis, which comprises 242 drug pairs with a fixed dose of imatinib, was performed and the highest selectivity achieved in these combinations was 2.8 for the combination of A15 and 17AAG plus imatinib (Table S4).

### Identification of optimal drug combinations using a search algorithm

We have developed a search algorithm that integrates two stochastic combinatorial optimization methods known as particle swarm optimization and genetic algorithm [18]. Initially, five doses of each single agent were considered and coded as number ‘0’ to ‘4’ (Table 2). The level 2 and 3 correspond to the low and high doses in the pair analysis, respectively. During algorithmic searches we realized that the doses used for some of the drugs were too high, resulting in combinations that were too toxic to the IMR-90 control cells. For other drugs the dose of maximal selectivity was not clear. Therefore after the third iteration, the concentration grid was reshaped to include two higher or lower levels depending on the drug performances (Table 2). After this reshaping of the drug concentration grid the algorithm was able to find highly selective combinations.

**Table 2:** Concentrations (µM) and levels of 11 small molecules used with imatinib in the combinatorial drug searches.

Level\Drug	A7	A12	A15	C16	E03	I22	P15	ABT263	Axitinib	17AAG	PD325901
1	0.025	0.125	0.05	0.0125	0.0125	0.00125	0.125	0.125	0.025	0.0025	0.005
2	0.05	0.25	0.1	0.025	0.025	0.0025	0.25	0.25	0.05	0.005	0.01
3	0.1	0.5	0.15	0.05	0.05	0.005	0.5	0.5	0.25	0.0125	0.025
4	0.25	1	0.25	0.1	0.1	0.01	1	1	0.5	0.025	0.05
**Level\Drug (Adjusted)**	**A7**	**A12**	**A15**	**C16**	**E03**	**I22**	**P15**	**ABT263**	**Axitinib**	**17AAG**	**PD325901**
1	0.025	0.125	0.05	0.0125	0.0125	0.00125	0.125	0.0125	0.025	0.0025	0.005
2	0.05	0.25	0.1	0.025	0.025	0.0025	0.25	0.05	0.05	0.005	0.01
3	0.1	0.5	0.15	0.05	0.05	0.005	0.5	0.125	0.25	0.0125	0.025
4	0.25	1	0.25	0.1	0.1	0.01	1	0.25	0.5	0.025	0.05
5	0.3	1.5	0.35	0.125	0.125	0.0125	1.25	0.5	0.55	0.03	0.06
6	0.35	1.75	0.45	0.175	0.175	0.0175	1.75	1	0.8	0.0425	0.085

The initial iteration was based on the 242 pair-wise measurements with imatinib (triplets) and a geometric average model that can estimate the selectivity of an arbitrary combination (see Methods). The search algorithm used these estimates to suggest the initial 270 combinations that were experimentally tested. After this initial iteration, the best selectivity was already improved by a factor of four compared to the best triplet selectivity of the previous step. (Table S4). At the sixth iteration, we had cumulatively tested 1607 combinations and the iterative search converged to an optimal combination set.

**Figure 3:**
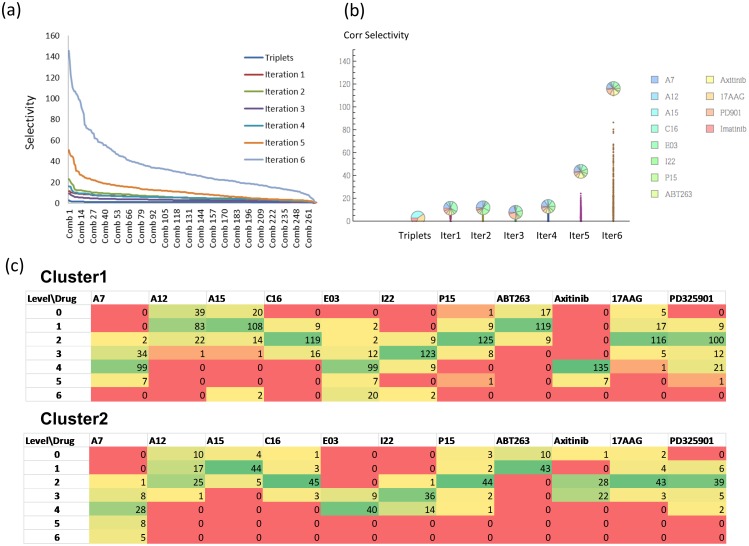
Iterative search for a highly selective drug combinations (a) Increased selectivity after each iteration step. The lines represent the selectivity of the drug combinations at as triplets and at each of the six iteration steps. (b) A dot plot showing corrected selectivity (see Results) through the iterative searches. The pie symbols shows the relative contributions of the 12 drugs for the best combination obtained at each iteration. (c) Cluster analysis on the optimized drug combinations. The top ranked 200 combinations clustered into two groups and the frequencies of the doses of the individual drugs are shown for each cluster.

Figure 3 illustrates the optimization of drug combinations. The function being maximized is selectivity. Note that after the third iteration, when the dose levels were adjusted, the selectivity drastically improved. In the last three iterations we also refined the selectivity measure using a “corrected selectivity” that further penalized combinations that are toxic to the IMR-90 control. The corrected selectivity was obtained through a multiplicative term 
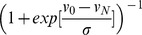
, where v_0_ is a cut off value describing the maximum allowed toxicity on normal cells, v_N_ is the viability of the normal cell, and σ is a smoothing parameter. We were able to attain a selectivity of 145 in the sixth iteration and a few combinations showed viabilities of more than 90% for IMR-90 control cells and of less than 2% for K562 cells (Table S4). The best combinations had relatively high doses of A7 and Axitinib and low doses of 17AAG and ABT263.

After the final iteration, we selected the top 200 combinations and performed a cluster analysis based on the euclidean distance metric. We observed two clusters; a larger cluster of 146 combinations and a smaller cluster of 54 combinations (Figure 3(c)). The major difference between two clusters is in the dose of Axitinib. The analysis of the top 200 optimized combinations also suggested the optimal dose ranges of the individual drugs. Most of the drugs were used at a concentration lower than 100 nM, which represents a significant reduction compared to the concentration range at which the single drugs were effective. For example, the highest selectivity we could achieve using single drugs was 12 with 0.05 µM 17AAG whereas the best combinations required a 10-fold lower dose of the same drug to reach 10-fold higher selectivity (Table S4).

### Confirmation of optimal drug combinations using an imatininb-resistant cell line and imatininb-resistant and sensitive primary patient cells

**Figure 4:**
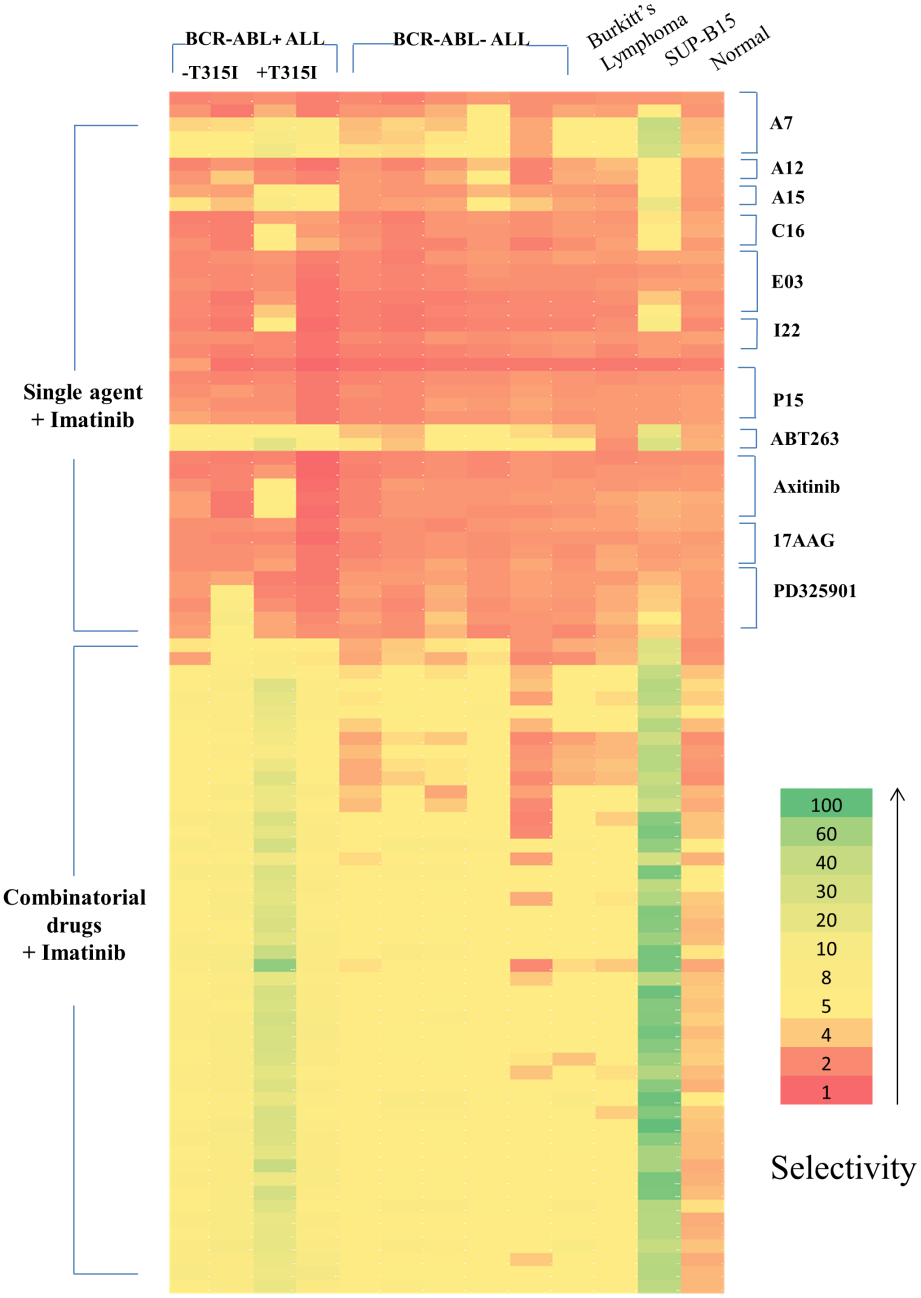
Test of optimum drug combinations in the patient cells. The best 49 drug combinations or single agents used for the combination were tested in the cells from BCR-ABL+ and BCR-ABL- ALL patients. The selectivity (vs control IMR fibroblasts) obtained in each patient specimen was color-coded from red (lowest) to green (highest). The drug combinations were also tested in Burkitt's lymphoma patient and in normal subject white blood cells as controls.

To demonstrate that our optimal combinations can also be applied to imatinib-resistant cells, we selected 49 drug combinations and tested them in the SUP-B15 cell line and ALL patient cells with and without the BCR-ABL translocation (Table S5). The combinations were selected using two criteria: assuring that the 49 combinations were representative of both cluster 1 and cluster 2 using a 70% viability of IMR-90 as cut-off and assuring that low toxicity combinations with 90% viability of IMR-90 as cut-off were included. Effective cell killing was also observed with the imatininb-resistant cell line, SUP-B15, which showed even higher selectivity than K562, assuring that drug combinations work well on both imatinib-sensitive and resistant cells. We were able to achieve a selectivity of 19 (B001) and 24 (B002) with selected drug combinations when tested in BCR-ABL+ ALL xenograft derived patient cells (Figure 4 and Table S6). In addition, two BCR-ABL+ patient-derived cells with the T315I mutation (B003 and B004) were included in order to determine if the combinations are also effective in known imatinib-resistant patient cells. Interestingly, higher selectivity was achieved with one of the imatinib-resistant patient cells (B003) but the other patient cells also responded well. The data shown in Figure 4 also demonstrate that several drugs and combinations we identified with K562 cell line are effective against BCR-ABL- ALL cells although the selectivity was not as high as for BCR-ABL+ ALL cells or cell lines.

A statistical analysis of the drug combination results shows that the mean differences between BCR-ABL+ ALL patients and normal subject are larger than those between ALL or Burkitt's lymphoma patients and normal subject (Table 3). The differences were statistically significant for all the BCR-ABL+ patient cells and for all but one of the BCR-ABL- patient cells. On the other hand, the single agents showed mostly negative mean differences, indicating that the single agents/imatinib pairs were generally not effective at the doses used for the combinations. Additionally, CD34+ hematopoietic stem cells (HSCs) were used to further study the toxicity of the drugs. We also observed high selectivities of BCR-ABL+ ALL cells against CD34+ HSC controls, although the viability of CD34+ cells tend to be lower than IMR-90 cells (Table S7).

**Table 3:** Results of statistical analysis using repeated measures one-way ANOVA with Dunnett's multiple comparisons between patient cells and normal mononuclear blood cells (N1) on viability of the best drug combinations originating from the cell lines search and from pairs of single agents plus imatininb.

Dunnett's multiple comparisons	Mean Diff. (combinations)	Adjusted P Value (combinations)	Mean Diff. (pairs)	Adjusted P Value (pairs)
N1 vs. B001	0.3468	<0.0001	−0.1379	0.0427
N1 vs. B002	0.4064	<0.0001	−0.195	0.077
N1 vs. B003	0.4570	<0.0001	0.6418	0.9174
N1 vs. B004	0.3884	<0.0001	−0.8320	<0.0001
N1 vs. B031	0.1914	<0.0001	−0.2496	<0.0001
N1 vs. B032	0.314	<0.0001	−0.304	<0.0001
N1 vs. B033	0.3054	<0.0001	−0.08711	0.121
N1 vs. B035	0.3132	<0.0001	−0.03637	0.951
N1 vs. B036	0.06369	0.2464	−0.1242	0.0043
N1 vs. B037	0.258	<0.0001	−0.05527	0.1199
N1 vs. Burkitt	0.1959	<0.0001	−0.03135	0.7161

Patients B001 and B002 are BCR-ABL+ (ALL). Patients B031 to B037 are BCR-ABL- (ALL).

ABT263 was, however, highly selective even as a single agent in leukemic cells although combinations always performed better than individual drugs (Table S6). Interestingly, the patient cells responded differently to some of the drugs. For example, only one patient (B002) responded to PD0325901 whereas the other patients did not respond to it, at least as a single agent. Primary cells isolated from Burkitt's lymphoma did not respond to the drug combinations as well as leukemic cells.

## Discussion

In this study, we have identified a list of potential drugs for BCR-ABL+ leukemias using three different approaches (kinase inhibitor screens, correlation analysis, and literature survey) and optimized the drug combinations using an iterative experimental search algorithm. Among the identified drugs, some showed additive effects when paired with the BCR-ABL+ targeting drug imatinib. Within only six iterations, we were able to find effective drug combinations that induced very selective killing of the leukemic cells (K562) versus the control fibroblast cells (IMR-90). The selectivities we achieved with the more effective drug combinations were significantly higher than those with the single agents, both in cell lines and in patient cells.

17-AAG is already known for its cytotoxic effects on imatinib-resistant patient cells and for its synergistic activities with imatinib in AML patient cells, in agreement with our correlation based analysis. [10], [19], [20] However, the doses of 17-AAG used in the previous combination study were much higher than those in the present study. [10] Some of the kinases we targeted in our combinatorial experiments, such as AKT and JNK, are known to be downstream of the BCR-ABL fusion protein. [21], [22] The BCL2 family inhibitor ABT-263 was highly potent in our study, selectively killing both K562 cells and leukemic patient cells at nM ranges. A previous study reported that ABT-737, an analogue of ABT-263, greatly enhances the apoptotic effect of INNO-406, a second-generation BCR-ABL inhibitor, against BCR-ABL positive leukemic cells. [23] In that study 17-AAG showed synergistic effects with ABT-263. The use of these two small molecules was suggested by our computational correlation analysis, but they or their analogues are already under clinical evaluation for the treatment of leukemia. [24], [25] In addition, a previously published report shows that subutoclax, a pan-BCL2 inhibitor, sensitizes leukemia stem cells to dasatinib treatment. [26] These literature results validate our methodology for selecting the set of anti-leukemic drugs to be used in the experimental searches.

The best drug combinations selected through the search with the K562 cell line also showed great efficacy in patient cells. The drug combinations were especially effective on BCR-ABL+ leukemic cells, but they also appeared to have less pronounced but still strong cytotoxic effects on several BCR-ABL- ALL patient cells (Figure 4). This is not unexpected, because they target proteins generally implicated in leukemia proliferation, part of common signaling cascades between BCR-ABL+ and BCR-ABL- leukemic cells. For example, it was previously reported that ABT-263 was not only able to induce cell death in BCR-ABL+ CML but also to induce complete remission of BCR-ABL- ALL cells in a mouse model. [24], [27] Although imatinib was used to target BCR-ABL, it has been reported that this compound also inhibits ARG, PDGFRA, PDGFRB, and c-KIT. [28], [29] Additionally, kinase profiling studies performed by Anastassiadis *et al*. show that many other kinases are targeted by imatinib. [30] Further studies will be required to understand in detail the mechanism of action of Imatinib in BCR-ABL- cells. In addition, the drug combinations also proved highly effective in the imatinib-resistant cell line, SUP-B15, [31] and in BCR-ABL+ leukemic cells with T315I mutation, pointing to the potential application of these drug combinations in tyrosine kinase inhibitor-resistant patients.

Our study presents a novel search algorithm for the identification of drug combinations that markedly increase the selective cell killing of leukemic cells. The highest selectivity achieved through iterative searches was not obtainable with a single agent or combination of two or three agents but required combining a larger number of drugs (see Table S4 and Figure 3). Whereas the current methodology to identify effective drug combinations often involves exhaustive testing, several papers have previously suggested approaches to combinatorial control using drugs based on biological search algorithms. [32], [33]. Our group has previously shown that algorithms derived from the field of digital communication can be used to find combinatorial therapies. [33]. Wong *et al*. developed a closed loop control algorithm using a microfluidic platform to implement an iterative stochastic search for optimal drug combinations. [32], [34], [35] In this study, we integrated particle swarm optimization and a genetic algorithm [18] to perform a biological search for drug combinations. We provide a detailed description and pseudocode in the methods section. Both methods are inspired by biological phenomena and have been very successful in multiple computational and engineering applications. [18]

The particle swarm algorithm mimics the behavior of a colony of insects that explore a field for food: each insect locally explores a section of the field, but there is exchange of information between them. Therefore, if an insect finds a region with abundant food, it will “inform” other insects and will attract them to that region. In our case, we consider a set of “agents” moving in the multi-dimensional space of all possible drugs and doses. Each agent explores a “neighborhood” of combinations, defined by adding and subtracting one dose. When the algorithm evolves, each agent has two options that are randomly chosen: (1) it can remain within its neighborhood and move towards the direction of the local optimal combination, or (2) it can look at how successful have been other agents and move towards neighborhoods of “more successful” agents.

The genetic algorithm is inspired by the concepts of crossover and mutation in natural selection. We can think of a “population” of drug combinations in which the genotype of each individual is represented by the sequence of doses corresponding to an ordered set of drugs. Top individuals are allowed to “mate” by swapping 50% of their dose sequence to generate new “children” combinations. A small probability of mutation is also added, meaning that the dose of a randomly chosen drug is modified. In our procedure, we first identified combinations according to the particle swarm method, and then, using the top 10% individuals, we generated “children” combinations until a maximum value determined by the number of plates available is reached.

The most effective combinations were composed of most of the drugs at low doses. These large combinations are analogous to the combinatorial strategies used by nature, as shown by the large number of regulatory molecules (e.g. transcription factors, kinases and microRNAs) that determine cell fate and differentiation. [12] Large drug combinations are also more likely to overcome or prevent cancer resistance, which is one of the main factor limiting the efficacy of targeted therapies in cancer [36] and they can potentially address intra-patient heterogeneity. It will be necessary to pursue further studies not only of efficacy but also of toxicity before their use in patients can be considered. In this study, we have included human mononuclear cells and CD34+ HSCs as a part of toxicity studies (Table S7). In future studies it might be desirable to further optimize drug combinations against a range of control cells to ensure low toxicity of the drug combinations. Our approach also holds promise for the identification of personalized therapies, some of them composed of only two dugs, as shown in Figure 4 in the case of the PD325901 and imatinib combination in one if the BCR-ABL+ patients.

Another challenge originates from the low predictive value of *in vitro* studies, even if performed with patient derived primary cells. [37] We have recently analyzed [38] the metabolomics of the human ALL bone marrow microenvironment. This might contribute, together with additional molecular studies addressing other components of this niche, for example cytokines, to the design of cell culture media that mimic more closely the patient conditions and accelerate the translation of our combinatorial therapy findings.

In summary, using an experimental search algorithm we have identified drug combinations including imatinib. These drug combinations are effective in a cell line and in primary cells and may provide a first-step toward finding combination therapies for the treatment of leukemic patients, especially if BCR-ABL+.

## Methods

### Ethical Statement

All clinical investigations were conducted according to Declaration of Helsinki principles. All human studies were approved by the UCSD Human Research Protections Programs IRB. Written informed consent was received from participants prior to inclusion in the study. Written informed consent and written parental permission were obtained in accordance with Institutional Review Board guidelines.

### Cell lines and patient cells

K562 and IMR-90 cells were maintained in RPMI (Invitrogen, USA) supplemented with 10% fetal bovine serum (FBS, Hyclone, USA), 100 U/ml penicillin, 100 µg/ml streptomycin (Hyclone, USA) at 37°C in 5% CO_2_. CD34+ hematopoietic stem cells were purchased from Allcells and maintained in StemSpan SFEM (STEMCELL Technologies, BC, Canada) with 100 ng of SCR, TPO, G-CSF (Life Technologies, CA, USA), and 1 µM of StemRegenin1 (EMD millipore, CA, USA).

Bone marrow specimens from ALL BCR-ABL- patients were obtained at the time of diagnosis from the Rady Children's hospital (San Diego, CA, USA). BCR-ABL+ ALL patient xenografts were obtained, as described in Bicocca *et al*., from the Muschen lab. [39] Diagnostic and cytogenetic information of the patients is included in Table S8. Blood from normal subjects was obtained from the Scripps Research Institute (San Diego, CA, USA). The study was performed in accordance with Institutional Review Board guidelines after obtaining informed consents from the patients. Briefly, mononuclear cells were isolated by Ficoll-paque (GE Healthcare, CA, USA) density gradient centrifugation (1.077 g cm^−3^) at 400 *g* for 30 min without brake followed by three washes in PBS. Patient xenografts and ALL patient mononuclear cells were subsequently cultured in RPMI with 10% FBS.

### Kinase inhibitor library screen

We identified candidate hits from the EMD kinase library (EMD Millipore, USA) screening at 1 µM concentration of kinase inhibitors with/without imatinib (0.125 µM). The values of relative cell survival from four replicates of two independent screens were averaged. Selectivity (v_n_/v_c_, where v_n_ and v_c_ indicate the viability of IMR-90 and K562) and imatinib combination index (v/v_imatinib_, where v and v_imatinib_ indicate the viability of K562 without/with imatinib) were calculated and rank-ordered from the highest to the lowest.

### Combinatorial studies

The combinatorial drug studies were done with the Echo automated liquid handler (Labcyte, CA, USA). Using our lab-developed high throughput screening manager software, the input files for combinatorial drug transfers were generated and drug droplets were transferred from the drug source plate to the designated wells of destination plates in 2.5 nl increments.

### Measurement of cell viability

Cell survival was assessed by luciferase-based assay, ATPlite (PerkinElmer, CA, USA), which determines viable cell numbers by measuring the presence of ATP in all metabolically active cells. For the measurement of cell viability, K562 and IMR-90 cells were plated at a density of 2000 and 1500 cells/384-well, respectively whereas primary patient cells were seeded at a density of 8000 cells/384-23ll. Subsequently, the cells were treated with the drugs and 72 hours later, the ATPlite assay was performed according to the manufacturer's protocol, and luminescence was read with an Analyst HT instrument (Molecular Devices, CA, USA). IC50 values were calculated from nonlinear regression analysis using Prism 6 (Graphpad).

### Gene expression correlation analysis

The Pearson correlation and p-values between mRNA expression and drug IC_50_ values for each gene were computed over 789 cell lines for 132 drugs [15]. This computation was repeated for all genes identified on the microarray. Next, the overlap of significant genes (p-value <0.001) between drugs and imatinib was computed and separated into four distinct comparisons. Given that four combinations of two drugs with either positive or negative correlations (namely negative-negative, negative-positive, positive-negative, and positive-positive correlation pairs) for a given gene are possible, we computed the number of shared genes for each possible drug pair and each of the four sign pairings. Each gene could receive only one correlation and corresponding sign (positive or negative) for a given drug. Since some drugs have a larger number of significant genes, we re-weighted the overlap parameter in order to get a normalized value. The formula
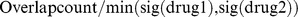
was used to re-weight the overlap between gene-to-drug combinations, where overlap is the number of genes meeting the 0.001 p-value significance cutoff that appear with desired sign pair (e.g. pos-pos) for the two drugs tested. The denominator is the minimum number of significant genes meeting the p-value cutoff for either of the two drugs tested individually.

### Initial conditions for the search algorithms

The initial set of multidrug combinations tested in the first iteration of the algorithm was obtained using results of the single agent and pair screening. Imatinib was included in all combinations at a fixed dose (0.125 µM) and we considered all the possible pairs of the other eleven drugs of Table 1 at the doses ‘0’ to ‘4’ corresponding to concentrations given in Table 2 (a). The selectivity of a multidrug combination can be described as the function
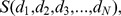
where *N* *= 11* and *d_i_* is the dose of the drug *i*. We can then estimate the selectivity function as a geometric average of the measurements in the single and pair screening as
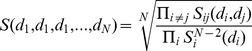
were 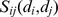 indicates the selectivity measured using only drugs *i* and *j* and 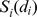
is the selectivity measured using only drug *i*. Note that since *d* can take the value ‘0’, these expressions include also the selectivity values measured with Imatinib alone and Imatinib with a second drug.

The estimates for all possible combinations based on this geometric average were then ranked and we used the top 10 combinations as initial position of the “particles” in the “particle swarm optimization” described below.

### Algorithmic method

The algorithm implemented in this search integrated a particle swarm optimization (PSO) method and a genetic algorithm (GA) [18]. We can think about the PSO in terms of a set of particles that move in the space of drugs and doses. The algorithm depends on two parameters, α and β that provide the relative contribution of the local and the global maximum to the velocity of the particles. We used 10 particles in our implementation and we set the maximum number of new combinations to be measured at each iteration to Max_comb = 270. The parameters of the algorithms were optimized through testing on simulated selectivity sets generated combining data from many previous multi-drug screenings on cell lines. The procedures for the PSO and GA implemented in the search are schematically described in BOX 1 and BOX 2. (Figure S2)

### Statistical analysis

Statistical analyses were performed using Prism 6 (Graphpad). Matched patient and normal subject specimens were compared using Dunnett's multiple comparison tests after repeated-measures analysis of variance (ANOVA).

## Supporting Information

Figure S1 (PDF)

Figure S2 (TIF)

Table S1 **Screen of EMD kinase inhibitor library.** Kinase inhibitors were rank-ordered according to the imatinib combination index (viability of K562/viability of K562 with imatinib). Top three kinase inhibitors were selected accordingly.
(XLS)

Table S2 **Correlation sign analysis between imatinib and drug responses.** Shared genes from positive/positive Pearson correlation between mRNA expression, imatinib IC50 values and mRNA expression, drug IC50 values.
(XLSX)

Table S3 **IC50 values of the selected kinase inhibitors.** IC50 values (µM) were calculated from the nonlinear regression analysis.
(XLS)

Table S4 **Rank ordered selectivity when algorithm-generated combinations were tested at each iteration.**
(XLS)

Table S5 **The list of top 49 combinations and corresponding single doses.** 49 drug combinations were selected using two criteria: top combinations with a 70% viability of IMR-90 and low toxicity combinations with a 90% viability of IMR-90.
(XLSX)

Table S6 **seletivities achieved from the selected combinations.** Selected combinations were tested in BCR-ABL+ and BCR-ABL- ALL patient cells and imatinib-resistant ALL cell line, SUP-B15.
(XLS)

Table S7 **seletivities achieved from the selected combinations.** Selected combinations were tested in BCR-ABL+ patient cells and CD34+ hematopoietic stem cells.
(XLSX)

Table S8 **Diagnostic and cytogenetic criteria of Patients.**
(XLS)
