# Supplementary figures and images for: Identification of Drug Combinations Containing Imatinib for Treatment of BCR-ABL+ Leukemias
Source: PLoS One. 2014 Jul 16;9(7):e102221. doi: 10.1371/journal.pone.0102221 (PMC4100887; doi:10.1371/journal.pone.0102221)

Relative cell survival

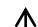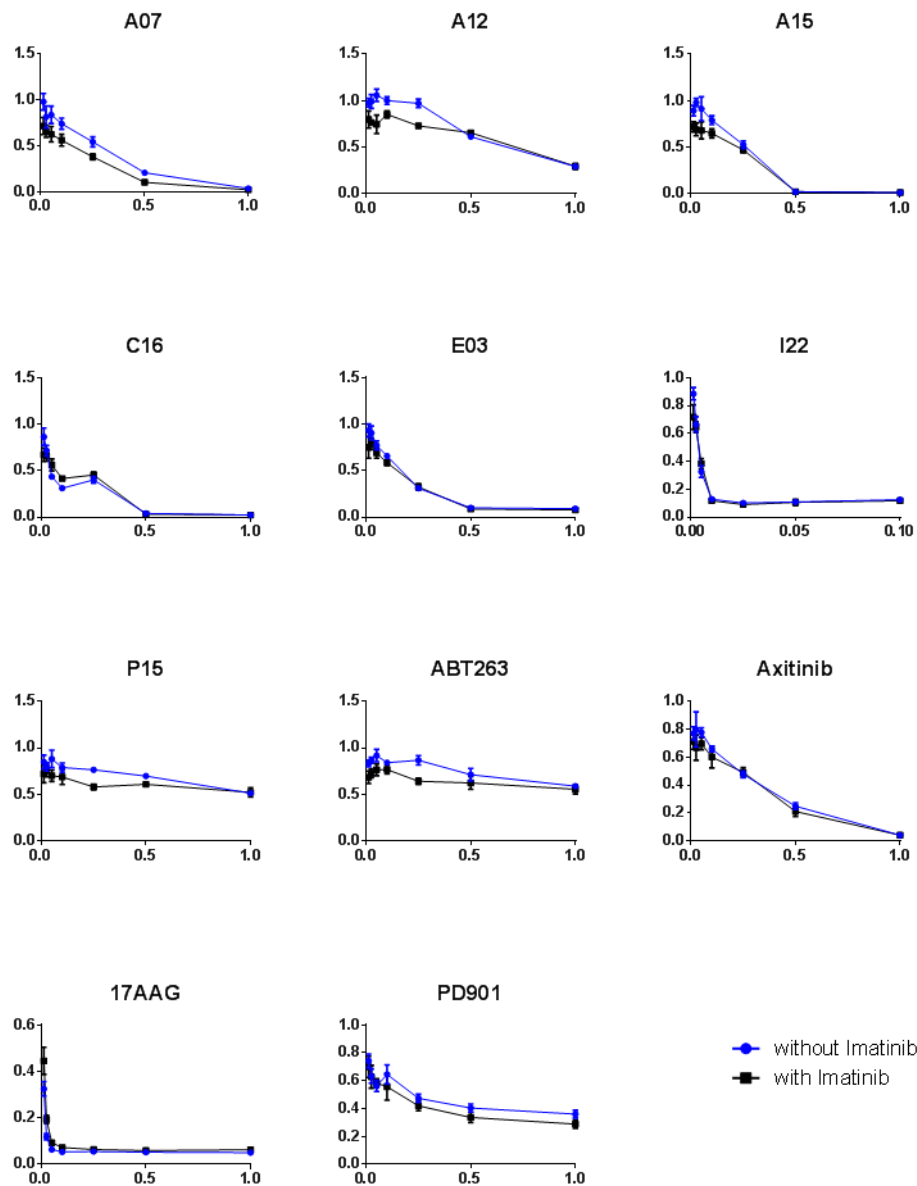

Doses ( $\mu\text{M}$ )

Supplement: Figure S1 — (PDF) [file pone.0102221.s001.pdf]

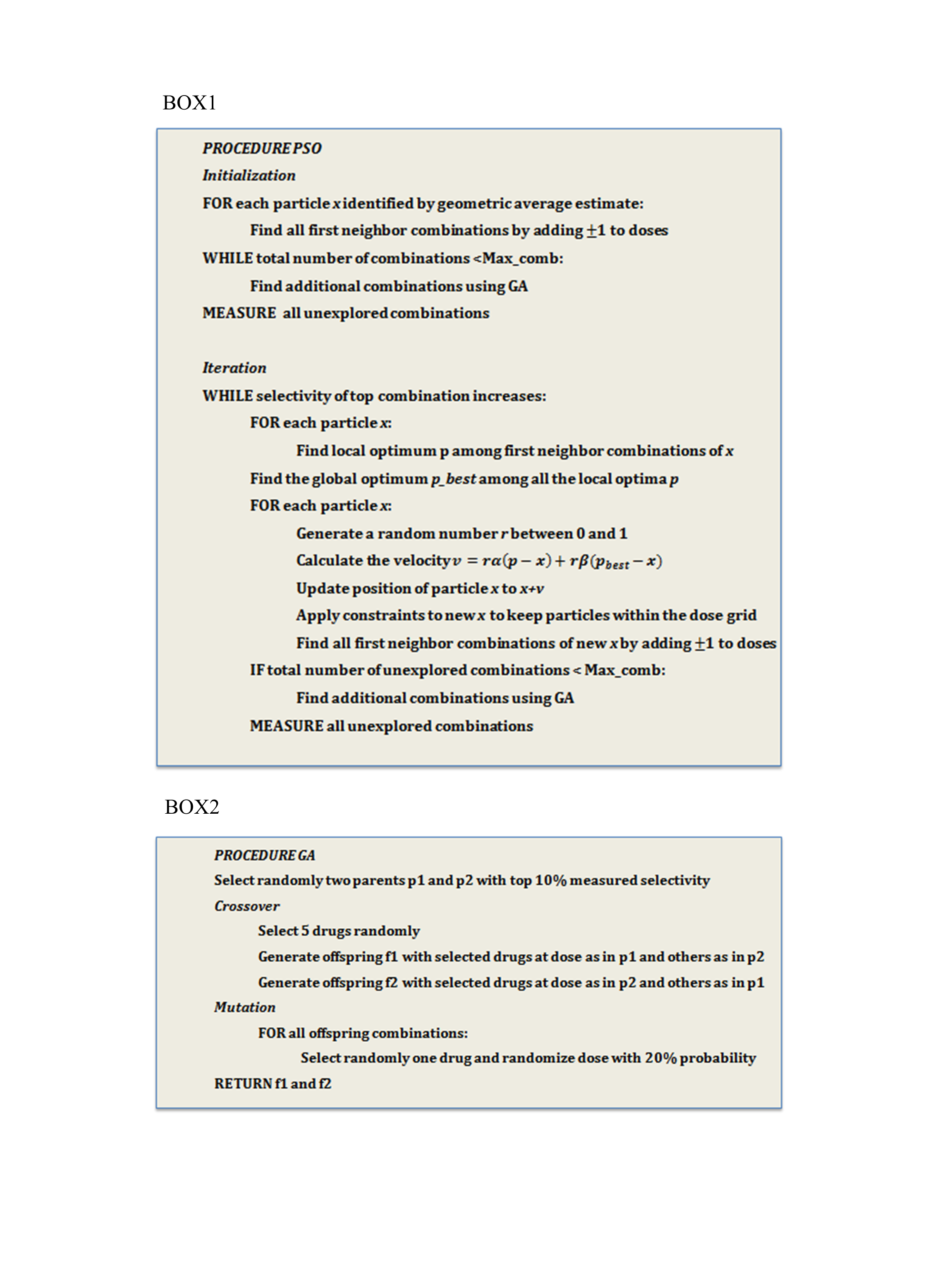

Supplement: Figure S2 — (TIF) [file pone.0102221.s002.tif]
